# Early Chest Re-Exploration for Excessive Bleeding in Post Cardiac Surgery Patients: Does It Matter?

**DOI:** 10.7759/cureus.15091

**Published:** 2021-05-18

**Authors:** Mujahid ul Islam, Imtiaz Ahmad, Bahauddin Khan, Azam Jan, Niaz Ali, Waasay Hassan Khan, Omer Farooq, Hooria Khan, Faizan Ahmad Ali, Muhammad Shahid

**Affiliations:** 1 Anesthesiology, Rehman Medical Institute, Peshawar, PAK; 2 Cardiothoracic Surgery, Rehman Medical Institute, Peshawar, PAK; 3 Cardiac Surgery, Northwest School of Medicine, Peshawar, PAK; 4 Radiology, Hayatabad Medical Complex, Peshawar, PAK

**Keywords:** chest reopening, excessive bleeding, surgical bleeding, coagulopathy, blood transfusion

## Abstract

Introduction

Re-explorations after open-heart surgery are often required if the patient is bleeding or shows features of cardiovascular instability and does not improve with conservative measures. Our study aims to determine whether timely re-exploration of patients who are bleeding has an impact on the morbidity and mortality of the patients.

Methods

A retrospective analysis of 75 patients that underwent open-heart surgery and subsequently underwent chest re-exploration for excessive bleeding between^ ^March 2018 and March 2020. Patients who were reopened post-op for indications other than excessive bleeding were excluded.

Results

A total number of cases were 700, out of which 75 (9.3%) patients were reopened, as compared to the literature, which shows worldwide 2-11% being reopened. Post-operative drain output was 1000ml to 1500ml in 47 (62.7%) and more than 1500ml in 28 (37.3%) patients before they were reopened. In 67 (89.3%) patients, three to five units of blood were transfused, and in eight (10.7%) patients, more than five units of blood were transfused. We believe our mortality in the reopened patients was low, because of timely intervention and early re-exploration, and is probably the reason why our figures land in a higher range (2-11%) of reopened cases (9.3%). Reopening time was less than five hours in 49 (65.3%) patients and less than 10 hours in 26 (34.7%) patients in our study. We tried to minimize the loss of blood and re-explored the patients before they lose excessive blood, the average time for reopening in our study was less than 10 hours. The average intensive care unit (ICU) stay was 4.2 days (range three to six days). Wound infections were reported in one of three patients. There was no mortality in these patients. Surgical site of bleeding was identified in 54 (72%) patients and no particular site was found in 21 (28%) patients. Suggesting that it is common to have a surgical bleeder than coagulopathy induced bleeding in post-cardiac surgery patients

Conclusions

We believe our low mortality (0%) is due to early reopening in patients who are bleeding excessively after cardiac surgery.

## Introduction

In post-cardiac surgery patients, excessive bleeding occurs in 2-11% of cases [1]. There are some recognized pre-disposing factors like advanced age, urgent or emergency procedures, poorly controlled diabetes, pre-operative low hemoglobin levels, elevated inflammatory markers, prolonged bypass time, complicated procedures, redo procedures, platelet dysfunction caused by antiplatelet drugs, and incomplete heparin reversal [1]. Coagulopathy and surgical bleeders have been associated with post-operative bleeding [2,3]. Post-operative bleeding is said to be excessive if it fulfills the Kirklin and Barratt-Boyes criteria, although various other endpoints are attempted to describe post-operative bleeding definitions.

Kirklin and Barratt-Boyes criteria

It includes (1) drainage of more than 500ml during the first hour, more than 400ml during each of the first two hours, more than 300ml during each of the first three hours, more than 1000ml in total during the first four hours, and more than 1200ml in total during the first five hours; (2) excessive bleeding that restarts (indicating a possible surgical cause); and (3) sudden massive bleeding.

It has been found in the previous studies that the mortality, intensive care unit (ICU) stay, and wound infection rate is found to be less in cases that had a surgical source of bleeding identified [1,4]. Post-operative excessive bleeding can be due to surgical bleeders or coagulopathy [2,3]. Excessive bleeding renders patients to increased morbidity and mortality [1,4]. Therefore timely intervention is expected to decrease the mortality rates, decrease ICU and hospital stay, and lower wound infection rates. Implementation of viscoelastic coagulation monitoring protocols has been shown to decrease coagulopathy-related bleeding [5]. Coagulopathy is usually multi-factorial; novel anti-platelet drugs, acidosis, bypass-related coagulopathy, heparin rebound, hypothermia, and more frequently surgical bleeding [6,7]. The source is found to be surgical in two-thirds of cases re-explored [6,8]. In either scenario, initial management is to replace blood loss, manage coagulation derangements with fresh frozen plasma and platelets, correction of hypothermia, manipulate inotropes to ensure adequate perfusion, and meanwhile keeping eye on trends of parameters like pulse rate, arterial pressure, central venous pressure, urine output, lactic acidosis, and hematocrit loss [8]. If despite these adequate conservative steps, there is deterioration in the parameters, re-exploration of the chest to control the bleeding is considered. In a previous study, it has been shown that the implementation of point-of-care hemostatic testing within the context of an integrated transfusion algorithm reduces red blood cell transfusions, platelet transfusions, and major bleeding following cardiac surgery [9]. Apart from immediate outcomes, long-term graft patency after coronary artery bypass grafting (CABG) is also reduced in patients who are re-opened for excessive bleeding. Early graft failure seems to be more frequent with the use of great saphenous veins, in poor target vessels and re-explored patients [10]. Direct trauma to anastomotic site, fresh frozen plasma (FFPs), platelet transfusions, and tranexamic acid are other possible contributors. Therefore the incidence of re angiography at one year is much higher in patients who are reopened [5,7]. Mortality after chest reopening is as high as 21%, 22%, 37%, in the published literature [1,6]. At the moment there is no standard recommended time for re-exploration [1,4]. Early re-exploration of the chest avoids blood and fluids transfusions, hypothermia, acidosis, renal injury, and acute respiratory distress syndrome (ARDS) [9]. The rationale of our study is to develop a time-based recommendation for chest re-exploration for excessive post-operative bleeding after cardiac surgery. We strongly believe that this would reduce mortality and morbidity rates.

## Materials and methods

Patients studied were operated in the Department of Cardiothoracic Surgery, Northwest General Hospital and Research Center, by the same team. The data of the targeted population of our study was extracted from a maintained electronic database at the cardiothoracic surgery department of a tertiary care hospital. The study was authorized by the research ethics committee. The data were analyzed using the Social Package for the Social Sciences (SPSS; Armonk, NY: IBM Corp.).

Inclusion criteria

A total number of 75 patients (both genders and all ages) who had open cardiac surgery and subsequently underwent chest re-exploration for excessive bleeding operated by the cardiac surgery team at Northwest General Hospital and Research Center between March 2018 and March 2020 were included in this study.

Exclusion criteria

Cardiac surgery patients who were re-opened post-operatively for indications other than excessive bleeding were excluded, to preserve the homogeneity of the sample population

Variable description

We evaluated the variables including excessive bleeding, re-opening, ICU stay, mortality, and wound infection. Excessive bleeding was defined as bleeding in pericardial and pleural drains as per Kirklin and Barratt-Boyes criteria. We analyzed the total number of days spent by the patients in ICU, based on the patient's clinical situation. Mortality was defined as the death of patients during the same admission. Wounds exhibiting clinical signs of inflammation along with discharge elevated total leukocyte count, and positive swab cultures were also analyzed. 

## Results

A total number of cases were 700, out of which, 75 (9.3%) patients were reopened, as compared to the literature, which shows worldwide 2-11% being reopened [4,7]. Among reopened cases, 60% were males and 40% females, 57.3% patients were re-explored on an urgent basis and 42.7% on a non-urgent basis. Low ejection fraction (<45%) patients constituted 49.3% of cases and the remaining (50.7%) had a good left ventricular function. Table 1 lists the general characteristics and pre-operative comorbidities of patients. 

**Table 1:** Patient characteristics and pre-operative comorbidities.

		N (%)	Count
Patient gender	Male	60.0	45
	Female	40.0	30
Diabetic status	Controlled	10.7	8
	Uncontrolled	84.0	63
	Non-diabetic	5.3	4
Hypertensive status	Controlled	30.7	23
	Uncontrolled	52.0	39
	Non-hypertensive	17.3	13
Patients on clopidogrel 75mg once daily	Taken more than 5 days ago	52.0	39
	Taken less than 5 days ago	48.0	36
Urgent procedure	Non-urgent	57.3	43
	Urgent	42.7	32
Ejection fraction	EF > 45%	49.3	37
	EF < 45%	50.7	38

Post-operative drain output was 1000ml to 1500ml in 47 (62.7%) and more than 1500ml in 28 (37.3%) patients before they were reopened. In 67 (89.3%) patients, three to five units of blood were transfused, and in eight (10.7%) patients, more than five units of blood were transfused. We believe our mortality (0%) in the reopened patients was low, due to early intervention and re-exploration of patients who were bleeding. Reopening time was less than five hours in 49 (65.3%) patients and less than 10 hours in 26 (34.7%) patients. We tried to minimize the loss of blood and re-explored the patients before they lose excessive blood. Our study reports no mortality in patients who were re-explored for bleeding. Table 2 mentions the post-operative parameters of the patients.

**Table 2:** Post-operative parameters. FFPs: fresh frozen plasmas; ICU: intensive care unit

		Count	N (%)
Total drain in ml before re opening	< 500 ml	0	0.0
	501-1000 ml	0	0.0
	1000-1500 ml	47	62.7
	> 1500 ml	28	37.3
Number of transfused blood bags	< 3 bags	0	0.0
	3-5 bags	67	89.3
	> 5 bags	8	10.7
	No transfusion	0	0.0
Number of FFPs bags transfused	< 3 bags	18	24.0
	> 3 bags	57	76.0
Number of platelets transfusion	< 3 bags	48	64.0
	> 3 bags	27	36.0
Reopening time in hours after shifting to ICU	< 5 hours	49	65.3
	6 - 10 hours	26	34.7
	> 10 hours	0	0.0
Extubation time after shifting in hours	0-5 hours	0	0.0
	6-10 hours	53	70.7
	11-15 hours	21	28.0
	16-20 hours	1	1.3
ICU stay in days	1-2 days	45	60.0
	3-5 days	30	40.0
	> 5 days	0	0.0
Wound infection	Wound not infected	65	86.7
	Wound infected	10	13.3
Mortality	No mortality	75	100.0
	Mortality	0	0.0
Surgical bleeding source identified	Surgical bleeder identified	54	72.0
	No particular bleeder identified	21	28.0

**Figure 1:**
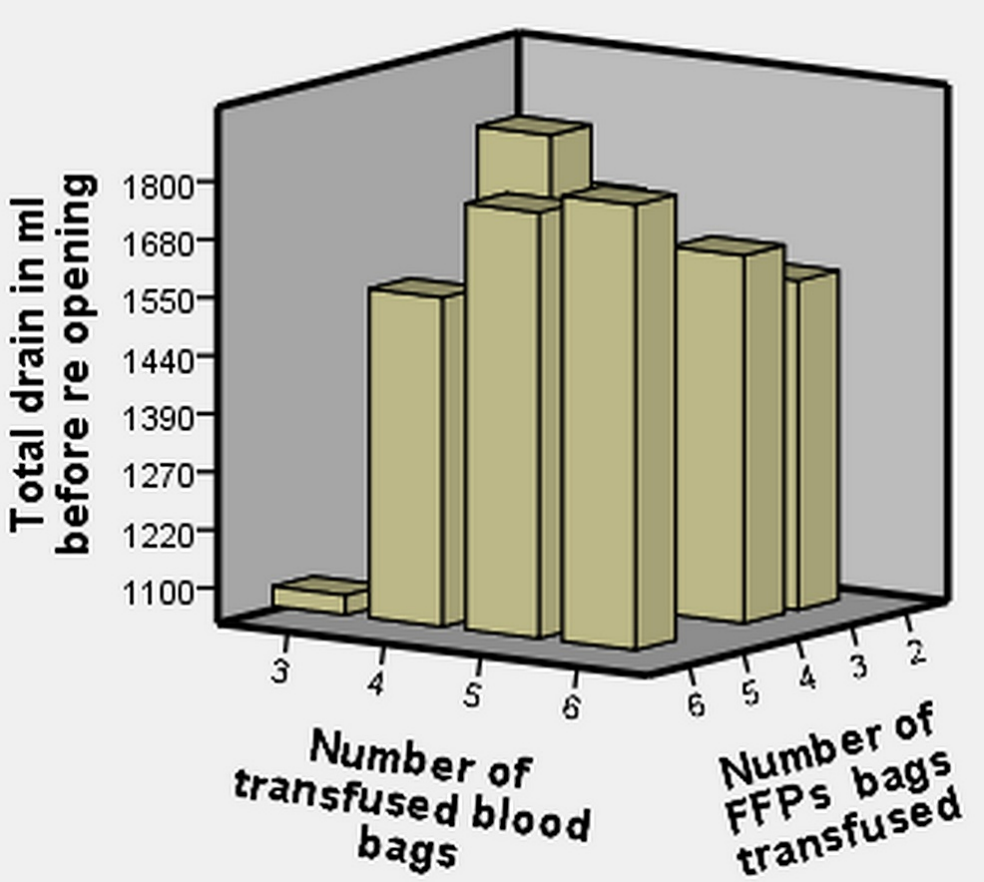
Blood products usage. FFPs: fresh frozen plasmas

Figure 1 illustrates the amount of blood products used according to the total amount of blood collected in the drain. Patients in whom the drain output was 1000ml to 1500ml received up to three to five units of blood and FFPs, and patients who had a drain output of more than 1500ml had received more than six units of blood and FFPs.

## Discussion

The Bleeding Academic Research Consortium (BARC) has defined significant coronary artery bypass graft-associated bleeding as chest tube output of greater than 2l in 24 hours, and transfusion of five or more units of packed red blood cells (PRBCs) in 48 hours [11]. Rarely, an actively bleeding patient can be taken to these endpoints and early steps to optimize the patient are often necessary. They are medically optimized with transfusion of blood and blood products and reoperation. This starts with pre-operative identification and if possible, optimization of risk factors.

Pre-operative risk factors include clopidogrel taken within five days of surgery, ticagrelor has taken within three days, patients receiving heparin for acute coronary syndromes, patients on warfarin or novel oral anticoagulants, and those on low molecular weight heparin. A cardiopulmonary bypass machine also affects hemostatic ability. Heparin rebound, hypothermia, and acidosis are other contributory factors. Surgical bleeders from the anastomosis site, left internal mammary artery (LIMA) bed, and extracardiac sites are other sources of excessive bleeding. Therefore lack of hemostasis is a multistep derangement that often coexists [8,12].

Steps were taken to decrease bleeding start with correction of hypothermia and acidosis. Occasionally, positive end-expiratory pressure (PEEP) of around 10cm may be used to tamponade small bleeders. Activated thromboplastin time, prothrombin time, platelet count, fibrinogen levels, complete blood count, and thromboelastography are often done to assess the status and cause of excessive bleeding [6,13]. Fresh frozen plasma, platelets transfusion, and occasionally desmopressin are given to enhance coagulation. Heparin rebound is treated with additional protamine. Recombinant factor VII is a novel agent used to treat coagulopathy. In addition, tranexamic acid and aminocaproic acid are also frequently used [7,10].

Reoperation is needed in 2.2-11.6% patients undergoing adult cardiac surgery [1,5]. Patients who require reoperation are at higher risk of kidney injury, cerebral complications, and sternal wound infections as well as increased mortality [1,9]. Literature shows causes of excessive bleeding were due to technical reasons (74%), coagulopathy (13%), a combination of coagulopathy and technical reasons (10%), and others (3%) [6,10]. Studies also reveal that most of the reoperations for bleeding are due to technical reasons rather than coagulation failures [4,14].

Timing of reoperation is also a matter of debate [8,15]. While it is important to correct hypothermia, acidosis, and coagulopathy before considering reoperation, it must be kept in mind that delay would result in increased morbidity and mortality. In a study by Choong et al. in 2007, of 3229 CABG patients, 157/191 were re-explored within 12 hours, and 34/191 were explored after 12 hours after cardiac surgery was completed. In the under 12 hour group, patients had a shorter ICU stay, less intra-aortic balloon pump (IABP) support, and lower mortality [8]. These findings were consistent with a study done prior to it [8,15]. Delay in reoperation exposes the patient to more transfusions and hence complications [8,15]. Another study states that the patients who only received blood transfusions as compared to patients who were re-explored for excessive bleeding had lower mortality and morbidity [16].

Different Intraoperative strategies are adopted to overcome bleeding, i.e., stitching, ligation, and clip application are common if a specific point is identified. In one study Fibrin sealant or topical hemostatic agent intraoperative were analyzed in 333 patients. 92.6% success was reported [17]. Delayed sternal closure is often used to avoid tamponade and later clot removal [18]. The application of vacuum-assisted closure is another option recently explored [19].

## Conclusions

In conclusion, excessive post-operative bleeding following cardiac surgery is a frequent occurrence. Acknowledging the multitude of factors leading to adverse outcomes, time of reoperation is an area less well explored. We attempt to fill this gap in the literature with our study. Despite a small number of patients and a single-centered study, findings in our study indicate that early re-exploration helps reduce the number of transfusions and the stress on the physiology of the patients thus improving the outcomes in terms of mortality and morbidity. We strongly recommend a multicenter prospective study to further evaluate our rationale.
